# Adjuvant or Salvage Radiation Therapy for Prostate Cancer after Prostatectomy: Current Status, Controversies and Perspectives

**DOI:** 10.3390/cancers14071688

**Published:** 2022-03-26

**Authors:** Mario Terlizzi, Elaine Johanna Limkin, Yasmina Moukasse, Pierre Blanchard

**Affiliations:** Radiation Oncology Department, Institute Gustave Roussy, 94805 Villejuif, France; elaine.limkin@gustaveroussy.fr (E.J.L.); yasmina.moukasse@gustaveroussy.fr (Y.M.); pierre.blanchard@gustaveroussy.fr (P.B.)

**Keywords:** prostate cancer, radiation therapy, adjuvant, salvage

## Abstract

**Simple Summary:**

The management of patients with biochemical recurrence after prostatectomy has undergone significant changes in recent years. Currently, close monitoring of prostate-specific antigen (PSA) with early salvage radiotherapy (RT) in case of recurrence is the standard of care based on several randomized trials and a meta-analysis that has demonstrated its non-inferiority to adjuvant RT. Uncertainties remain regarding the management of patients at very high risk of recurrence, including appropriate selection criteria for adjuvant hormone therapy, and the role of imaging in refining the treatment strategy. This review explains this paradigm shift, raises points of controversy, and suggests ways to think about the future.

**Abstract:**

Nearly one-third of the patients who undergo prostatectomy for prostate cancer have a biochemical recurrence (BCR) during follow-up. While several randomized trials have shown that adjuvant radiation therapy (aRT) improves biochemical control, this strategy has not been widely used because of the risk of toxicity and the fear of overtreating patients who would not have relapsed. In addition, the possibility of close PSA monitoring in the era of ultrasensitive assays enables to anticipate early salvage strategies (sRT). Three recent randomized trials and their meta-analysis have confirmed that aRT does not improve event-free survival compared to sRT, imposing the latter as the new standard of treatment. The addition of androgen deprivation therapy (ADT) to RT has been shown to improve biochemical control and metastasis-free survival, but the precise definition of to whom it should be proposed is still a matter of debate. The development of genomic tests or the use of artificial intelligence will allow more individualized treatment in the future. Therapeutic intensification with the combination of new-generation hormone therapy and RT is under study. Finally, the growing importance of metabolic imaging (PET/CT) due to its performance especially for low PSA levels will help in further personalizing management strategies.

## 1. Introduction

Prostate cancer is currently the second most frequent malignancy in men worldwide, responsible for nearly 360,000 deaths each year [1]. Radical prostatectomy (RP) +/− pelvic lymph node dissection (PLND) is one of the cornerstones of treatment for localized diseases but almost 20–40% of patients all stages combined will present with biochemical recurrence (BCR) in the first 10 years after surgery, requiring additional radiation therapy (RT) to be cured [2]. RT can either be delivered as an adjuvant treatment, based on the assessment of the risk of recurrence using prostatectomy histological criteria, or as a salvage treatment at the time of the BCR. As BCR precedes the appearance of metastases with a mean time of 8 years and specific mortality of about 15 years, this constitutes a window of opportunity that should not be missed from a curative perspective [3]. In recent years, several studies have changed the paradigm so that the indications for adjuvant therapy have drastically declined. 

This review explores currently available evidence guiding the choice between adjuvant and salvage RT, the addition of androgen deprivation therapy (ADT) to RT, the place of advanced imaging, and future avenues for research. Data from published randomized controlled trials or large retrospective studies were used to summarize the available data. This allowed a broad analysis of the literature but didn’t exclude selection bias.

## 2. Are All Patients at Equal Risk of Biochemical Relapse?

There is a broad spectrum of patients treated with RP, with variable prognoses. Several pathological factors have been associated with increased risk of BCR after RP: a high Gleason score (≥8), pT3a (extracapsular extension) or pT3b (seminal vesicle invasion), positive margins (R1), and lymph node invasion (N1). A combination of these criteria further increases the risk of BCR. In a series by Abdollah et al., aRT improved survival only in men having less than two factors among Gleason score ≥ 8, pT3b/T4 stage and pN1 stage [4].

The PSA remains to be the most reliable biomarker of disease persistence or recurrence. However, for a long time, there was no universal consensus in the cut-off value defining BCR. A PSA > 0.4 ng/mL was described as the threshold best associated with the development of distant metastases in the post-operative setting [5]. The relevance of this threshold to guide postoperative RT has decreased in the era of the ultrasensitive PSA assay, and since it has been shown that the lower the PSA was at the time of RT, the higher biochemical control and lower distant metastasis (DM) rates [6]. Currently, BCR after RP is defined according to the European Association of Urology (EAU) guidelines as two consecutive PSA values ≥ 0.2 ng/mL [7]. Beyond the absolute value of the PSA, its kinetics (evaluated by the doubling time, PSA-DT) has also a prognostic value [8]. Antonarakis et al. showed in a retrospective series of 450 men treated with RP that PSA-DT was an independent predictive factor of MFS [9].

In order to take into consideration the variability of possible clinical scenarios and assist in decision making the 2019 EAU-ESTRO-ESUR-SIOG guidelines proposed a useful BCR classification system stratifying patients into low (PSA-DT > 1 year and pGS < 8 after RP) or high-risk of BCR (PSA-DT ≤ 1 year or pGS 8–10). This stratification was externally validated by a large cohort of 1125 patients in which the BCR risk grouping was found as an independent predictor for DM (HR 3.46; *p* < 0.001) and prostate cancer-specific mortality (PCSM) (HR 5.12; *p* < 0.001) in multivariate analysis. Moreover, the effect of sRT was maximal for PSA level < 0.5 ng/mL arguing in favor of early delivery (HR 0.32 vs. 0.56 for DM and HR 0.31 vs. 0.58 for PCSM for PSA < 0.5 ng/mL and PSA ≥ 0.5 ng/mL, respectively) [10].

## 3. Radiation Therapy in the Post-Prostatectomy Setting: A Question of Timing

The assessment of the appropriate timing of postoperative RT has been the subject of several large trials that can be separated into older trials that compared aRT to surveillance [11,12,13,14] and newer trials that compared aRT to early sRT [15,16,17].

Four randomized trials have assessed the impact of aRT: SWOG 8794, EORTC 22911, ARO 96-02, and FinnProstate [11,12,13,14] (Table 1). Globally, patients with a high risk of relapse based on pathological criteria (pT3 ± positive margins) were randomized between aRT (total dose of 60–66 Gy in the prostate bed) or observation. At 10 years median follow-up, these studies have shown an increase in biochemical control of nearly 20% in favor of aRT. Only the oldest trial (SWOG 8794) has shown a benefit in terms of MFS and overall survival (OS) (HR 0.71; 95% CI 0.54–0.94; *p* = 0.016 and HR 0.72; 95 CI 0.55–0.96; *p* = 0.023 respectively) [11]. There are however several limitations that should be considered, and caution should be exercised when applying the results in clinical decision making. Populations were quite heterogeneous among studies. Only the ARO 96-02 required an undetectable PSA for inclusion (PSA < 0.1 ng/mL) whereas nearly 30% of patients in the SWOG 8794 and EORTC 22911 trials presented with a measurable PSA at inclusion and were therefore in an early salvage setting. Conversely, some patients in the observation arm did not receive sRT despite having measurable PSA. In a recent meta-analysis combining data from these four trials (n = 2068), Bhindi et al. reported a significant benefit in favor of aRT in terms of biochemical progression-free survival (bPFS) and local recurrence-free survival (HR 0.47; 95% CI 0.41–0.54 and HR 0.54; 95% CI 0.39–0.73, respectively). However, the benefit of MFS remains uncertain (mainly driven by the SWOG 8794 trial) and there is no OS benefit (HR 0.79 and 0.90, respectively) [18]. Two counterarguments cautioned against aRT and have led to its limited applicability in clinical practice. First, about one-third of patients in the observation arm did not have biochemical relapse despite their initial pathologic factors: this indicates that further treatment is avoidable for a large proportion of patients. Second, RT increases the rate of GU and GI toxicities when delivered early after RP [19]. In the EORTC 22911 trial, a higher rate of late any grade adverse event was reported in the aRT arm (cumulative incidence of 70.8% vs. 59.7% at 10 years; *p* = 0.001) [12]. Taken together, this suggests that certain patients would not benefit from treatment and could be exposed to unnecessary toxicities.

The treatment strategy has undergone a recent paradigm shift. Indeed, three randomized trials have compared aRT and early sRT at BCR in the era of PSA monitoring after RP: RADICALS-RT, RAVES, and GETUG-AFU 17 [15,16,17] (Table 2). These trials differed in design, inclusion criteria, PSA threshold triggering sRT, and primary endpoint measured. Considered separately, they showed no significant difference in terms of bRFS (biochemical recurrence-free survival) in delaying RT at biochemical relapse versus aRT. Moreover, long-term toxicity rates were more commonly reported in the aRT group. The collaborative and prospective ARTISTIC meta-analysis collated data from these three trials before the publication of their respective results. In order to homogenize the interpretation, a harmonized definition of event-free survival (EFS) was defined as the time without either BCR (PSA ≥ 0.4 ng/mL and rising after RT), clinical/radiobiological progression, initiation of a non-trial treatment, PSA ≥ 2 ng/mL at any time after randomization or death. The analysis included 2153 men. After a median follow-up ranging from 60 to 78 months among the studies, the EFS was not different between arms (HR 0.95; 95%CI 0.75–1.21; *p* = 0.70; 5-years EFS of 89% versus 88%). Interestingly, nearly 60% of men randomized in the sRT arm did not receive treatment, confirming that a wait-and-see strategy is reasonable in this patient population [20]. These recent data have contributed to recommending sRT as the standard treatment after prostatectomy, even in high-risk patients.

## 4. Is Adjuvant Radiation Therapy Definitively over?

Since the publication of the last three studies and the ARTISTIC meta-analysis, a surveillance strategy based on regular PSA testing with early sRT is the current standard, and aRT is no longer considered by radiation oncologists regardless of post-RP histological features. However, it should be noted that few of the patients included in these trials had highly unfavorable criteria and that these results are likely not applicable to the very high-risk subset of patients (e.g., pT3b Gleason 8–10 patients). Another limitation is that PSA recurrence was the endpoint of these studies, which is not an ideal surrogate for survival and may have been artificially prolonged by the use of ADT in the case of the GETUG-AFU 17 trial [17]. On the basis of a possible immortal time bias, Tilki et al. assessed the impact of aRT versus early sRT on all-cause-mortality (ACM) risk for the subset of men with pN1 or pGS 8–10 and pT3/4 stages. After a median follow-up of 8 years, aRT significantly reduced the ACM risk versus sRT among men with adverse pathology with or without the inclusion of pN1 (HR 0.66; *p* = 0.04 and HR 0.33; *p* = 0.02 respectively) [21]. Prospective studies are eagerly awaited to confirm these findings. Nonetheless, these data are retrospective and sRT should remain standard even in the high-risk population.

## 5. RT Plus ADT: Is It Necessarily a Winning Combo?

Since the combination of ADT and RT has been shown to be beneficial in terms of biochemical control and OS for patients with localized unfavorable intermediate and high-risk prostate cancer, the question of its use in the adjuvant/salvage setting is a matter of interest. However, uncertainties remain regarding patient selection, the duration and form of therapy, and the characteristics of the associated RT. The question has not been widely explored in the context of adjuvant treatment. Only retrospective series have suggested better biochemical control with ADT, particularly for patients at high risk of relapse [22,23].

Two randomized trials have assessed the impact of ADT on RT in the salvage setting. In the placebo-controlled RTOG 9601 trial, 760 patients were randomized between sRT alone versus sRT combined with 24 months of daily bicalutamide. The 12-year OS was increased by 5% in the bicalutamide group (HR 0.77; 95% CI 0.59 to 0.99; *p* = 0.04). Twelve-year MFS and cancer-specific survival (CSS) were also significantly higher among patients having received ADT (9.6% and 7.6%, respectively, *p* < 0.01) [24]. The second trial (GETUG AFU-16) randomized 743 patients between sRT ± 6 months of a GnRH agonist (goserelin). The updated results after a median follow-up of 112 months demonstrated a significantly higher 10-year biochemical and clinical PFS in the RT + ADT group (64% vs. 49%; HR 0.54, 95% CI 0.43–0.68; *p* < 0.0001). The benefit was also described in MFS (75% vs. 69%; HR 0.73, 95% CI 0.54–0.98; *p* = 0.034) [25]. It is important to note that it is difficult to compare the results of these two trials as populations were not similar. In the GETUG-AFU-16 trial, only patients with undetectable post-operative PSA were enrolled (whereas nearly 12% of patients in the RTOG 9601 had a measurable PSA at inclusion) and patients globally harbored more favorable profiles (with lower pre-RT PSA and lower pT3 stages). The decision to initiate ADT concurrently with RT must be carefully weighed against its potential side effects. In a recent secondary analysis of the RTOG 9601 trial, Dess et al. demonstrated the prognostic value of pre-RT PSA level on oncological outcomes: ADT was associated with a 12-year OS improvement for patients with PSA > 1.5 ng/mL (25% versus 1% for these with PSA ≤ 1.5 ng/mL). For the subgroup of patients with PSA ≤ 0.6 ng/mL, not only did ADT provide no OS benefit, it also tripled the risk of developing a grade 3 to 5 neurological or cardiac late event and doubled the risk of treatment-related mortality [26].

Several accruing trials will help better define the duration of concurrent ADT. The LOBSTER phase 2b trial (NCT04242017) will compare 6 versus 24 months of triptorelin both combined with sRT (70 Gy/35) for men experiencing BCR after pN0 RP.

## 6. Discussion and Perspectives

### 6.1. Will the Future Come from Metabolic Imaging?

The place of imaging remains debated in the post-operative setting and is often left to the discretion of the radiation oncologist. The main interest of imaging is as a tool to improve patient selection and be able to offer the most appropriate radiation treatment in terms of technique, fields, and dose; to improve oncological outcomes. Conventional imaging (bone scan and computed tomography) is classically unsuccessful in detecting sites of failure at low PSA levels in the setting of BCR. Several studies have indeed suggested that they both should be considered only in the case of high PSA levels (>10 ng/mL), high velocity, or for symptomatic patients [27,28]. In a previous study by Okotie et al., patients with PSA < 10 ng/mL after RP had positive findings on CT and bone scan in 0% and 11%, respectively (vs 57% and 46% for men with PSA > 10 ng/mL) [29]. For the detection of local recurrences, multiparametric MRI (mpMRI) of the pelvis has proven its accuracy, although it remains dependent on PSA levels [30].

Metabolic imaging with PET/CT has widely proven superior diagnostic performances for lower PSA. Several radiotracers—including choline, fluciclovine, and PSMA- are available. Positive findings of PET-choline are still dependent on the PSA level: the detection rate decreases from 76% to 20% for PSA decreasing from >2 to <1 ng/mL [31]. Pooled sensitivities and specificities of choline-PET were found to be 85% and 88% for local recurrence and 100% and 82% for nodal relapse, respectively [32,33]. Conversely, PSMA-PET/CT is currently the metabolic imaging that offers the best diagnostic performance for low PSA levels. In a retrospective series of 119 patients with a PSA < 0.5 mg/mL, ^68^Ga-PSMA-11 PET/CT demonstrated a detection rate of nearly 35%, surpassing the performance of PET/CT with other radiotracers [34]. A large systematic review and meta-analysis of 4790 patients by Perera et al. identified a strong correlation between increasing PSA levels and the probability of detection of distant relapse using ^68^Ga-PSMA. In the post-prostatectomy cohort, the proportions of positive imaging were 33%, 46%, 75%, 82%, and 97% for PSA categories 0–0.19, 0.2–0.49, 0.5–0.99, 1–1.99 and ≥2 ng/mL, respectively [35]. Several studies have evaluated the effect of metabolic imaging on RT planning. Specifically, in the context of BCR, the findings of ^68^Ga-PSMA-11-PET/CT induced change on the planned RT in 20 to 60% of cases [34,36,37,38,39] (Table 3). Calais et al. reported a prospective head-to-head comparison between ^18^F-fluciclovine-PET/CT and ^68^Ga-PSMA-11-PET/CT in 143 patients with low PSA concentrations (<2.0 ng/mL) in the BCR context. The metastasis detection rate was lower with ^18^F-fluciclovine-PET/CT than with ^68^Ga-PSMA-11-PET/CT (26% versus 56%; OR 4.8, 95%CI 1.6–19.2; *p* = 0.0026). The authors concluded that PSMA should be the preferred radiotracer for decision making in the BCR context [40].

In order to take all these elements into consideration, some authors have proposed algorithms for decision making. Based on clinicobiological features (time to biochemical relapse since surgery, PSA and PSA-DT) and pathological features (Gleason score, status margins, and pT/pN stages), a risk analysis can be carried out and lead to mpMRI in the case of low risk of metastases or metabolic imaging in the case of a suspected distant recurrence (the radiotracer is then chosen according to the PSA level) [41]. It is only recently that the impact of such imaging on oncological outcomes has begun to show promising results. The EMPIRE-1 (Emory Molecular Prostate Imaging for Radiotherapy Enhancement) trial was the first randomized phase 2/3 trial to assess the impact of metabolic imaging exclusively on post-prostatectomy relapsing patients. Patients with detectable PSA after RP and with negative conventional imaging were randomized between RT directed by conventional imaging or by conventional imaging combined with fluciclovine-PET/CT. In the PET/CT arm, RT decisions and planning followed the imaging findings: RT was canceled in the case of extra-pelvic uptake, maintained with adapted treatment fields in the case of prostate bed ± nodal uptake, and finally focused only on the prostate bed if no uptake. The 3-year event-free survival was significantly higher in the PET-directed RT arm (75.5% vs. 63%, *p* = 0.0028) and toxicity rates were similar between groups [42]. Currently, the EMPIRE-2 trial (NCT 03762759), randomly assigning patients to fluciclovine-PET/CT or PSMA-PET/CT RT directed, is recruiting. Whether treatment adaptations induced by metabolic imaging will influence survival outcomes remains a challenging question.

The main question that currently remains open is what strategy to adopt in the case of negative findings on PET imaging. A wait-and-see attitude with repeated imaging until a target is identified is a highly interesting proposal, as it would have the advantage of sparing RT for patients without proven relapse and then offering salvage treatment with appropriate doses and treatment fields in the case of a macroscopic target (instead of doses of about 60–65 Gy in a large volume). This strategy could be proposed for highly selected patients: in a retrospective study about 103 men with negative PSMA-PET/CT at BCR and followed up for at least one year, Celli et al. reported a higher clinical recurrence-free survival in case of PSA < 0.5 ng/mL and low-grade cancer on RP specimens (ISUP 1 and 2) [43]. Conversely, a study by Emmett et al. showed that PSMA-PET/CT was predictive of 3-year PFS for men treated by sRT. While a negative finding on PSMA-PET/CT was a more important predictor of long-term sRT control than other risk factors, this could indicate that such patients even benefit from prostate bed RT. Finally, 34% of men with negative imaging did not receive sRT and were free of relapse at three years, suggesting the possibility of close monitoring in a selected subgroup [44]. In the EMPIRE-1 trial, salvage RT to the prostate bed was delivered even in the case of no uptake on PET/CT.

There is no current robust consensus regarding the place of metabolic imaging in the setting of biochemical relapse, but further changes are to be expected. The current EAU guidelines suggest the use of PSMA-PET/CT or choline/fluciclovine-PET/CT in the case of PSA > 0.2 and PSA > 1 ng/mL, respectively, and if an impact on the management strategy is expected [45].

### 6.2. Towards A Personalization of Treatments

Recent developments in genomics may offer additional tools in the individualization of treatments, by identifying the subset of patients at high risk of recurrence eligible for intensified therapy. Most of the current data concerns the Decipher test (Decipher Biosciences, San Diego, CA, USA), a 22 gene-genomic classifier (GC) developed in the post-prostatectomy setting and having shown its predictive value in distant metastases (DM) [46]. Several validation series have demonstrated their superiority in their accuracy and prognostic value in comparison to clinical and pathological models [47,48]. Feng et al. recently published an ancillary study where Decipher was generated from 352 patients enrolled in the RTOG 9601 trial with a 13-year median follow-up. After adjusting for several clinical and pathological parameters, the Decipher test was independently associated with DM (HR 1.17; 95% CI 1.05–1.32; *p* = 0.006), PCSM (HR 1.39; 95% CI 1.20–1.63; *p* < 0.001), and OS (HR 1.17; 95% CI 1.06–1.29; *p* = 0.002) in multivariable analysis. Interestingly, in patients having received sRT for PSA < 0.7 ng/mL, the addition of ADT was beneficial in terms of DM and OS only in the case of intermediate-to-high GC (versus low GC) [49]. These data would therefore allow further refinement of the therapeutic strategy and the indications for concurrent ADT. Finally, promising preliminary data have shown that artificial intelligence-derived pathology-based biomarkers could help in predicting the benefit of ADT for localized stages. Its place in the post-operative setting represents a large area to be explored [50].

### 6.3. Is Drug Intensification the Key?

Studies of patterns of relapse after post-prostatectomy RT have shown that the first site of recurrence was mainly distant but that 1/5 of patients had an in-field relapse [51]. This indicates the need to improve both locoregional control (and thus overcome radioresistance mechanisms) and distant control (by eradicating the micrometastatic cells). Since new-generation hormone therapies have proven their benefit in increasing survival for metastatic and more recently for high-risk localized stages, their relevance in the salvage setting is a current challenge. Their use in concomitance with RT seems promising, with in vitro series suggesting enhanced radiosensitization of some androgen receptor inhibitors compared to ADT [52]. The phase 2 STREAM study was the first to investigate the combination of enzalutamide concurrently with RT and ADT. The 2-year PFS was 65% and a 29% toxicity grade 3 rate was reported [53]. The French randomized phase 3 CARLHA-2/GETUG-AFU 33 trial (NCT04181203) comparing prostate bed and pelvic RT with 6 months of ADT ± apalutamide for high-risk relapsing patients is currently recruiting. Other therapies targeting the Pi3K/Akt/mTOR pathway or glucose metabolism (such as metformin) are under investigation [54]. Finally, the use of therapeutic PSMA radiopharmaceuticals, such as Lu-PSMA, will also be of interest in this population with microscopic residual disease (Table 4).

## 7. Conclusions

For several years, the timing of post-prostatectomy RT has been the subject of intense debate. The current data argue in favor of delivering early sRT, when BCR occurs (PSA > 0.2 ng/mL on two consecutive assays). This strategy results in improved biochemical outcomes while sparing patients who have not had a recurrence from potential toxicities. However, survival data are not sufficiently mature and long-term results are needed to confirm the strategy. Some uncertainties remain, such as the appropriate selection of patients requiring concomitant ADT, and its duration. Intensified therapy strategies involving new molecules such as new-generation hormone therapy are being currently evaluated. Finally, the growing importance of metabolic imaging, in particular PSMA-PET/CT, and genomic data should allow more personalized therapeutic strategies in the future.

## Figures and Tables

**Table 1:** Randomized trials assessing adjuvant RT for prostate cancer.

Trial, Year	n	Inclusion Criteria	Arms	Median Follow-Up (Years)	% of Patients with Salvage RT for BCR in the Observation Arm	Median PSA at Salvage RT (ng/mL)	10 Year-bPFS	10 Year-MFS	10 Year-OS
SWOG 8794 (2009)	431	pT3 cN0 ± R1	60–64 Gy vs. observation	12.6	33%	0.75–1.0	60.7% vs. 47.4% (*p* < 0.005)	71% vs. 61% (*p* = 0.04) *	74% vs. 66% (*p* = 0.023)
EORTC 22911 (2012)	1005	pT2 pN0 R1 pT3 pN0 ± R1	60 Gy vs. observation	10.6	43%	1.7	60.6% vs. 41.1% (*p* < 0.001) *	89.9% vs. 89% (ns)	80.7% vs. 76.9% (ns)
ARO 96-02 (2014)	388	pT3 pN0 ± R1 undetectable PSA	60 Gy vs. observation	9.3 (adjuvant group), 9.4 (observation)	NR	NR	56% vs. 35% (*p* = 0.005) *	84.3% vs. 85.1% (ns)	82% vs. 86% (ns)
FinnProstate Group (2019)	250	pT2 R1, pT3a	66.6 Gy vs. observation	9.3 (adjuvant group), 8.6 (observation)	86%	0.7	82% vs. 61% (*p* < 0.001) *	98% vs. 96% (ns)	92% vs. 87% (ns)

Abbreviations: RT = radiation therapy, BCR = biochemical recurrence, bPFS = biochemical progression-free survival, MFS = metastasis-free survival, OS = overall survival, * indicates the primary endpoint of the trial, ns = no significance.

**Table 2:** Randomized trials comparing salvage and adjuvant RT.

Trial	Design	Patients Randomized	Inclusion Criteria	Trigger for Salvage RT	RT Schedule	ADT	Median Follow-Up (Months)	BRFS (aRT vs. sRT)	GU Late Toxicity (aRT vs. sRT)	GI Late Toxicity (aRT vs. sRT)
RADICALS-RT	superiority	1386	R1 or pT3a/T3b/T4 or Gleason 7–10	PSA ≥ 0.1 ng/mL or 3 consecutive rises	66 Gy/33 or 52.2 Gy/20	no	60	85% versus 88% (*p* = 0.56)	G3,4 haematuria 4% vs. <1% (*p* < 0.0001) G3,4 urethral stricture 6% vs. 4% (*p* = 0.0025)	G1,2 diarrhea and proctitis 13–17% vs. 5–8% (*p* < 0.0001) G3,4 (ns)
RAVES	non-inferiority	333	R1 or pT3a/T3b	PSA ≥ 0.2 ng/mL	64 Gy/32	no	73	86% versus 87% (*p* = 0.15)	≥G2 70% vs. 54% (*p* < 0.001)	≥G2 14% vs. 10% (ns)
GETUG-AFU 17	superiority	424	R1 and pT3a/T3b/pT4	PSA ≥ 0.2 ng/mL	66 Gy/33	yes	47	92% versus 90% (*p* = 0.42)	≥G2 59% vs. 22% (*p* < 0.0001)	≥G2 8% vs. 5% (*p* = 0.24)

Abbreviations: RT = radiation therapy, ADT = androgen deprivation therapy, Gy = Gray, BRFS = biochemical recurrence-free survival, GU = genito urinary, GI = gastrointestinal, PSA = prostate-specific antigen, ns = no significance.

**Table 3:** Selected studies assessing the impact of ^68^Ga-PSMA-11-PET/CT findings on the salvage RT planning.

Author, Year	Population	Median PSA (ng/mL) (Range)	Overall Detection Rate (%)	Extra-Pelvic Uptake (%)	RT Planning Change among Positive PET/CT (%)
Habl, 2017	100	1.0 (0.12–14.7)	76	7	59
Calais, 2018	270	0.48 (0.03–1)	49	12	19 (post-hoc)
Farolfi, 2019	119	0.34 (0.20–0.50)	34.4	21	30.2
Boreta, 2019	125	0.40 (0.28–0.63)	53	38	30
Bottke, 2021	76	0.25 (0.07–0.5)	54	8	28

**Table 4:** The management of BCR after RP: an overview of current status and trends.

		PSA Recurrence (>0.2 ng/mL on Two Consecutive Tests)	
		no	yes
Adverse pathological factors	no	PSA monitoring (SOC)	early salvage RT (SOC)
	yes	Adjuvant RT for highly selected cases?	
Areas of research and future development			
Advanced imaging			
Therapeutic intensification (ADT ± NHT)			
AI-guided management: genomics/pathomics			

Abbreviations: RT = radiation therapy, SOC = standard of care, ADT = androgen deprivation therapy, NHT = second-generation novel hormonal therapy, AI = artificial intelligence
